# Considerations for Postacute Rehabilitation for Survivors of COVID-19

**DOI:** 10.2196/19462

**Published:** 2020-05-08

**Authors:** Lisa Mary Sheehy

**Affiliations:** 1 Bruyère Research Institute Ottawa, ON Canada

**Keywords:** covid-19, rehabilitation, subacute care, inpatient rehabilitation, public health, infectious disease, virus, patient outcome, geriatric, treatment, recovery

## Abstract

Coronavirus disease (COVID-19), the infection caused by severe acute respiratory syndrome coronavirus 2 (SARS-CoV-2), was first reported on December 31, 2019. Because it has only been studied for just over three months, our understanding of this disease is still incomplete, particularly regarding its sequelae and long-term outcomes. Moreover, very little has been written about the rehabilitation needs of patients with COVID-19 after discharge from acute care. The objective of this report is to answer the question “What rehabilitation services do survivors of COVID-19 require?” The question was asked within the context of a subacute hospital delivering geriatric inpatient and outpatient rehabilitation services. Three areas relevant to rehabilitation after COVID-19 were identified. First, details of how patients may present have been summarized, including comorbidities, complications from an intensive care unit stay with or without intubation, and the effects of the virus on multiple body systems, including those pertaining to cardiac, neurological, cognitive, and mental health. Second, I have suggested procedures regarding the design of inpatient rehabilitation units for COVID-19 survivors, staffing issues, and considerations for outpatient rehabilitation. Third, guidelines for rehabilitation (physiotherapy, occupational therapy, speech-language pathology) following COVID-19 have been proposed with respect to recovery of the respiratory system as well as recovery of mobility and function. A thorough assessment and an individualized, progressive treatment plan which focuses on function, disability, and return to participation in society will help each patient to maximize their function and quality of life. Careful consideration of the rehabilitation environment will ensure that all patients recover as completely as possible.

## Introduction

Coronavirus disease (COVID-19), the infection caused by severe acute respiratory syndrome coronavirus 2 (SARS-CoV-2), was first reported on December 31, 2019. Because it has only been studied for just over three months, our understanding of this disease is still incomplete, particularly its sequelae and long-term outcomes. Knowledge about COVID-19, including its presentation and treatment, is changing very rapidly, and guidelines are quickly being created and updated. Therefore, it is important to remain current by engaging in frequent reviews of new research.

The objective of this report was to answer the question “What rehabilitation services do survivors of COVID-19 require?” The question was asked within the context of a subacute hospital delivering geriatric inpatient and outpatient rehabilitation services. As of April 14, 2020, very little has been written about the rehabilitation needs or outcomes for patients with COVID-19 after discharge from acute care. Upon thoughtful consideration of the question, it appears that the topics of greatest importance to allied health professionals treating patients with COVID-19 are the physical, cognitive, and psychosocial presentation of survivors, the procedures that would be required within a rehabilitation department, and the treatment that should be provided. These three topics are discussed in order below.

Much of what has been published is based on expert opinion but not on direct observation of the actual trajectories of patients with COVID-19. Many of the early papers came from China and Italy , the locations that had the earliest experience with COVID-19; these can potentially provide insight into longer-term outcomes and ongoing patient needs. Organizations such as the World Health Organization (WHO) and physiotherapy organizations have also written acute-care clinical practice guidelines for patients with COVID-19 [1,2]. Some authors have extrapolated based on postacute patient presentations and the rehabilitation needs of patients with similar conditions, such as severe acute respiratory syndrome (SARS), Middle East respiratory syndrome (MERS), and sepsis, and from those requiring intensive care unit (ICU) care and assisted mechanical ventilation for other reasons [1-7]. These suggestions have been included here, and research on these conditions has informed what follows here regarding patient presentation and rehabilitation. However, the physical presentations of SARS and MERS are different than that of COVID-19, and the experiences of patients with these diseases are not necessarily the same as those of COVID-19 patients. SARS mainly causes respiratory symptoms along with diarrhea, while MERS causes more gastrointestinal and kidney symptoms along with respiratory symptoms [4,8]. COVID-19 appears to cause a wider variety of symptoms that are related to many body systems (eg, cardiac, kidney, and nervous systems) [4,9-12]. SARS and MERS are more lethal than COVID-19, with fatality rates of approximately 10% and 36%, respectively, and patients with both diseases are more likely to be hospitalized and require mechanical ventilation [8].

## Patient Presentation For COVID-19 Survivors in the Rehabilitation Unit

Comorbidities, direct lung damage from COVID-19, and concurrent injuries to other organs and systems due to COVID-19 are all important considerations when creating a rehabilitation treatment plan for patients recovering from COVID-19. The information below presents several comorbidities and features of COVID-19; however, this knowledge continues to evolve.

### Comorbidities

The leading comorbid conditions of patients with COVID-19 are hypertension (55%), coronary artery disease and stroke (32%), and diabetes (31%) [10]. Patients with COVID-19 are less likely to have the following chronic illnesses: liver diseases (9%), chronic obstructive pulmonary disease (7%), malignancy (6%), chronic renal failure (4%), gastrointestinal diseases (3%), central nervous system diseases (<1%), and immunodeficiency (1%) [10]. Therefore, survivors requiring prolonged rehabilitation are more likely to be older and to have preexisting cardiovascular and cerebrovascular disease, which may influence their rehabilitation and outcomes.

### Complications of Severe COVID-19

The most likely early complications are acute respiratory distress syndrome (ARDS) and sepsis/septic shock, multi-organ failure, acute kidney injury, and cardiac injury [2,10,13]. These complications contribute to the need for ICU admissions [10].

Critical illness polyneuropathy (CIP) is a mixed sensorimotor neuropathy that leads to axonal degeneration; it may occur after COVID-19 [5,14-16]. In one study of patients hospitalized in the ICU with ARDS, up to 46% of patients presented with CIP [15]. CIP causes difficulty weaning from mechanical ventilation, generalized and symmetrical weakness (distal greater than proximal, but including diaphragmatic weakness), distal sensory loss, atrophy, and decreased or absent deep tendon reflexes [15,16]. It is associated with pain, loss of range of motion, fatigue, incontinence, dysphagia, anxiety, depression, posttraumatic stress disorder (PTSD), and cognitive loss [15]. Muscle biopsies and electromyographic testing can be diagnostic [15,16]; however, it is unclear how often these tests are performed in acute care settings post–COVID-19.

Critical illness myopathy (CIM), which presents in 48%-96% of ICU patients with ARDS, is a non-necrotizing diffuse myopathy with fatty degeneration, fiber atrophy, and fibrosis [5,15,16]. It is associated with exposure to corticosteroids, paralytics, and sepsis. The clinical presentation is similar to CIP but with more proximal than distal weakness and sensory preservation [15,16]. For both CIP and CIM, the cranial nerves and facial muscles are preserved [16]. Patients recover from myopathy more completely and quickly than from polyneuropathy; however, with both conditions, weakness, loss of function and quality of life, and poor endurance may persist for up to two years or even longer [15,16]. These prolonged changes are out of proportion with any residual loss of pulmonary function. Research studies on the effects of postacute care rehabilitation are inconclusive but suggest that comprehensive integrated inpatient rehabilitation is required [14].

Post–intensive care syndrome is described separately from CIP and CIM; it is associated with reduced pulmonary function (restrictive pattern), reduced inspiratory muscle strength, poor knee extension, poor upper extremity and grip strength, and low functional capacity [17]. Improvement occurs over a year or more [17].

### Potential Persistence of SARS-CoV-2 Virus

Patients with COVID-19 who have physically recovered and have tested negative for the virus twice are deemed to be cured and noninfectious. However, there are reports of such patients subsequently testing positive 5-13 days later using a different manufacturer’s test kit [18]. The virus may also persist in a patient’s oropharyngeal cavity and stools for up to 15 days after they are declared cured of COVID-19 (no fever, no respiratory symptoms, 2 negative swab tests) [19]. This is of particular concern for patients who are intended to be discharged to rehabilitation facilities or long-term care because they may still be able to transmit disease, potentially infecting other patients or residents. Because of this, an additional 14 days in quarantine or discharge to a dedicated COVID-19 step-down unit has been recommended [18,20].

### Cardiac Sequelae

In one study [13], 20% of hospitalized patients in China with COVID-19 had associated cardiac injury. These patients were more likely to have comorbidities, require mechanical ventilation, and have other complications (eg, ARDS 59%, acute kidney injury 9%, electrolyte disturbances 16%, hypoproteinemia 13% and coagulation disorders 7%) [13]. They also had much higher mortality (51% vs 5%) [13]. The mechanism of cardiac injury is uncertain [13]. Presentations can include arrhythmia, cardiac insufficiency, ejection fraction decline, troponin I elevation, and severe myocarditis with reduced systolic function [4,11]. One brief report profiled a woman with acute myopericarditis/heart failure post–COVID-19 [9]. As the research investigating cardiac injury included either cross-sectional studies or cohort studies with short-term follow-up (4 weeks), long-term outcomes are unknown [4,21]. Persistent tachycardia was common after SARS; however, it tended to resolve itself and was not associated with increased risk of death [4,13]. The presence of cardiac injury and accompanying comorbidities must be taken into consideration for patients entering rehabilitation.

### Neurological Sequelae

Acutely, 36.4% of patients with COVID-19 develop neurological symptoms, including headaches, disturbed consciousness, seizures, absence of smell and taste, and paresthesia [5,21]. Posterior reversible encephalopathy syndrome, which causes headache, confusion, seizures and visual loss, is a potential complication of COVID-19 [5]. Viral encephalitis has been reported to be caused by COVID-19, and brain tissue edema and partial neuronal degeneration have been found in deceased patients [12,22]. It is hypothesized that COVID-19 can increase one’s risk for acute cerebrovascular events [12]. At least one person has had Guillain-Barré syndrome associated with COVID-19; however, no causal relationship was determined [23].

SARS can induce neurological diseases such as polyneuropathy, viral encephalitis, and aortic ischemic stroke [24]. In MERS, almost one-fifth of patients showed neurological symptoms (altered consciousness, paralysis, ischemic stroke, Guillain-Barré syndrome, infectious neuropathy, or seizures) [25,26].

### Other Body Systems

Patients severely affected by COVID-19 are more likely to have acute kidney injury as well as secondary infection [10,11]. Survivors of ARDS with mechanical ventilation have reported complications such as tracheal stenosis, heterotopic ossification, contractures, adhesive capsulitis, decubitus ulcers, hoarseness, tooth loss, sensorineural hearing loss, tinnitus, brachial plexus injuries, and entrapment neuropathies (peroneal and ulnar) [7,15]. They also had concerns regarding scarring and changes in appearance due to a variety of causes [15].

Osteoporosis and avascular necrosis have been reported as sequelae of SARS [27]. These conditions may have arisen due to the use of corticosteroids, which are not a suggested treatment for COVID-19 [10]. The prevalence of the use of corticosteroids to treat COVID-19 in different cities and countries is unknown.

### Cognitive Sequelae

In one study of patients with respiratory failure or shock, after ICU admission (91% were mechanically ventilated), median global cognition scores (measured by the Repeatable Battery for the Assessment of Neuropsychological Status) were an average of 1.5 SD below the age-adjusted population mean and similar to those of patients with mild cognitive impairment [28]. Among these patients, 26% had scores 2 SD below the population mean, similar to scores for patients with mild Alzheimer disease [28]. Repeat testing at 12 months did not show much change [28]. The trend was the same for patients regardless of their age [15,28]. Cognitive impairment can persist [15,28]. Cognitive impairment can affect 70%-100% of patients at discharge; 46%-80% still have it one year later, and 20% still have it after 5 years [15]. All components of cognition can be affected, including attention, visual-spatial abilities, memory, executive function, and working memory [15,28]. However, there is a great deal of variation in these effects.

### Psychological Sequelae

In research regarding ICU admissions for ARDS, adverse psychological impacts have been reported [15]. Even after 2 years, PTSD (22%-24%), depression (26%-33%), and general anxiety (38%-44%) are prevalent [15]. These have been reported as concerns post–COVID-19 as well, accompanied by a severe reduction in quality of life and function [7]. One of the greatest risk factors for post-ARDS mood disturbances is premorbid psychiatric illness [15]. Other risks include younger age, female sex, unemployment, alcohol use, and greater use of opioid sedation [15]. Family members may also suffer from PTSD, anxiety, and depression, and they may have difficulty managing their new caregiver roles [15].

## Suggested Procedures for Post–COVID-19 Rehabilitation

After discharge from acute care, some patients who have recovered from the acute respiratory effects of COVID-19 will need further rehabilitation. How many of these patients may need postacute care? In one study, 30% of patients hospitalized with sepsis (which has a similar mortality rate to COVID-19) required facility-based care; another 20% required home health care [29].

### Design and Procedures for an Inpatient Rehabilitation Unit

These suggestions regarding the design of an inpatient rehabilitation unit in this time of COVID-19, and the procedures to be followed, are mostly based on the experiences of China and Italy, who are ahead of Canada on the COVID-19 trajectory [5,21,29-31]. Experience during the SARS epidemic has also informed these suggestions on the provision of rehabilitative care [32]. Considerations for the design and procedures for inpatient rehabilitation after COVID-19 will become more refined as more survivors are treated and facilities learn from experience. Each suggestion from the literature [5,21,29-31], stated below, needs to be evaluated based on the unique circumstances of each rehabilitation unit as well as the needs of the patients and the greater health care community.

- A separate unit or area is suggested for the rehabilitation of patients post–COVID-19 and other patients arriving on the unit.
- Depending on need, it has been suggested that dedicated facilities should be used to treat patients post–COVID-19; examples may include underutilized rural hospitals or retrofitted unused buildings, such as university dormitories.
- It may be necessary to receive patients from acute care earlier than is generally done.
- Patients should stay in their rooms.
- Group therapy and therapy in rehabilitation gyms should be prohibited; therapy should be provided one-on-one in patients’ rooms.
- Patients may be discharged to home sooner than usual (as soon as the family is able to take care of the patient) to free space.
- It may be difficult to discharge some patients because long-term care facilities and retirement homes may not be accepting new residents.
- Shared equipment must be decontaminated between patients; single-use equipment should be used where possible (eg, TheraBands rather than hand weights). Particular attention should be paid to electrode sponges, hydrocollator heat packs, gels, topical lotions, items for training manual dexterity, etc.
- Plan therapeutic activities to minimize the number of personnel involved when possible (eg, one therapist with a gait aid rather than a therapist and an assistant).
- Minimize the number of personnel entering a patient’s room. Have a single staff member perform most (if not all) of the care and duties for a particular patient (eg, deliver food trays, make the bed, give medication, help with morning care).
- Walking practice should be done in parts of the hospital that are not commonly used.
- Surgical masks should be worn by the patients and the therapists.
- Patients should be kept at least 2 meters apart and avoid talking or eating while facing each other.

### Personnel Considerations

Several suggestions for how allied health care professionals can adapt to working with COVID-19 rehabilitation patients are provided here. These suggestions have been informed by early COVID-19 reports and adapted from acute care guidelines [5,21,30,31].

- Health checks for personnel should be done frequently.
- There may be personnel shortages due to staff illness, staff in isolation, or redeployment.
- There may be changes in staff/patient ratios due to the increased number of one-on-one treatments (due to patients not being seen in the rehabilitation gyms).
- Continuous staff training will be required due to changing protocols/guidelines.
- Time should be taken to train and retrain personnel in the use of personal protective equipment (PPE).
- Physiotherapists and speech-language pathologists should wear higher levels of PPE if they may be exposed to aerosols from post–COVID-19 patients (eg, chest physiotherapy and swallowing assessments).
- It is important to seek ongoing input from front line staff to inform others. One group of rehabilitation professionals in Italy has been holding weekly webinars to stay up-to-date with the changing needs of rehabilitation during this time. These are available for an international audience.
- All nonrequired therapies and services should be cancelled, or telecommunication should be used to deliver them.
- The time taken to don PPE and perform infection control measures may decrease work efficiency.
- Allied health professionals should wear scrubs and a T-shirt at work and shower and change into street clothes before going home.
- Rehabilitation staff may be divided into two teams who work independently of each other. If several members of one team become ill, the other team can take over.
- Meetings should be held virtually when possible.

### Home-Based Rehabilitation

If patients can be managed at home, this may be a good option, even for patients who might have been admitted to inpatient rehabilitation in the past [29,32]. Isolation is easier at home, and the burden on inpatient services would be lessened [29,32]. However, for this to be a viable choice, enhanced homecare services and outpatient rehabilitation must be available and able to provide a level of care on par with inpatient rehabilitation. This mode of delivery may be difficult to institute if home care staff are restricted from entering patients’ homes [33]. However, given the right precautions, home-based care may be safer for patients who have recovered from COVID-19 and for other patients in a rehabilitation unit [33]. Home-based therapy can be provided over the internet and telephone via telerehabilitation [28]. Both assessment and treatment may be provided, either synchronously (ie, in real time) or asynchronously (eg, a prerecorded customized exercise plan). It is important that processes are put in place to ensure that patients and therapists can use this method successfully, given the rehabilitation needs and comfort with technology of the individual patient. One or more in-person visits may be required as well. Telerehabilitation may also be a good choice for patients being discharged from inpatient rehabilitation to continue their treatment and promote further recovery [30,32].

## Rehabilitation Guidelines After COVID-19

The importance of rehabilitation after COVID-19 has been emphasized according to the framework of the International Classification of Functioning, Disability and Health [34,35]. The WHO does not have rehabilitation guidelines for patients post–COVID-19 [2]; however, the situation is evolving quickly. Each patient should be fully assessed by all health care staff (physicians, nursing, and allied health care workers), and a suitable treatment plan should be created in conjunction with the patient and the team while considering the patient’s wishes and goals. The direct impact of COVID-19 (eg, on the respiratory system and other systems), its sequelae (eg, ICU stay, mechanical ventilation), and its comorbidities (eg, hypertension, diabetes) will inform the treatment plan [3]. The discharge destination and estimated discharge date will also affect the plan. What follows are some guidelines suggested by health care professionals in China, Italy, and other areas based on their experiences and expert opinions [3,6]. The guidelines are influenced by the prevailing rehabilitation in the regions; however, there is very little actual research on the impact of rehabilitation after COVID-19, with only one randomized controlled trial published to date [36].

### Respiratory Rehabilitation

Recommendations from both China and Italy state that to avoid aggravating respiratory distress or dispersing the virus unnecessarily, respiratory rehabilitation should not begin too early [3,37,38]. In the acute phase, diaphragmatic breathing, pursed lip breathing, bronchial hygiene, lung expansion techniques (positive expiratory pressure), incentive spirometry, manual mobilization of the ribcage, respiratory muscle training, and aerobic exercise are not recommended [37]. Secretions are not commonly a problem after COVID-19; however, comorbid conditions such as bronchiectasis, secondary pneumonia, or aspiration may increase secretions [7]. Postural drainage and standing (for gradually increasing periods of time) are suggested for secretion management [39].

In inpatient rehabilitation, respiratory assessment should include dyspnea, thoracic activity, diaphragmatic activity and amplitude, respiratory muscle strength (maximal inspiratory and expiratory pressures), respiratory pattern, and frequency [38,39]. Cardiac status should also be assessed [39].

In the postacute phase, inspiratory muscle training should be included if inspiratory muscles are weak. Deep, slow breathing, thoracic expansion (with shoulder elevation), diaphragmatic breathing, mobilization of respiratory muscles, airway clearance techniques (as needed), and positive expiratory pressure devices can be added based on assessed needs [38,39]. Care must be taken to avoid overloading the respiratory system and causing distress [7]. One randomized controlled trial showed a significant improvement in respiratory function, endurance, quality of life, and depression from 2 sessions of 10 minutes of respiratory rehabilitation per week for 6 weeks following discharge from acute care [36]. Rehabilitation included respiratory muscle training with a positive expiratory pressure device, cough exercises, diaphragmatic training (using 1 to 3 kilograms of weight on the abdomen in supine), chest stretching, and pursed-lip breathing. Patients should be monitored closely for shortness of breath, decreased SaO_2_ (<95%), blood pressure <90/60 or >140/90, heart rate >100 beats per minute, temperature >37.2 ºC, excessive fatigue, chest pain, severe cough, blurred vision, dizziness, heart palpitations, sweating, loss of balance, and headache [3,38].

### Mobility and Functional Rehabilitation

Functional assessment should include muscle joint range of motion, strength testing, and balance (use of the Berg Balance Scale is suggested) [3,7,38]. Exercise capacity can be assessed with the 6-minute walk test (with continuous oxygen saturation monitoring) and cardiopulmonary exercise testing. Function and disability can be measured with the International Physical Activity Questionnaire, Physical Activity Scale for the Elderly, and the Barthel Index to measure activities of daily living (ADLs).

Physiotherapy should begin in the acute inpatient setting and continue after transfer to inpatient rehabilitation [3,38]. Early mobilization should include frequent posture changes, bed mobility, sit-to-stand, simple bed exercises, and ADLs, while respecting the patient’s respiratory and hemodynamic states [1,7]. Active limb exercises should be accompanied by progressive muscle strengthening (suggested program: 8-12 repetition-maximum load for 8-12 repetitions, 1 to 3 sets with 2 minutes rest between sets, 3 sessions a week for 6 weeks) [3,38]. Neuromuscular electrical stimulation can be used to assist with strengthening. Aerobic reconditioning can be accomplished with overland walking, cycle or arm ergometry, or a NuStep cross trainer [7]. Initially, aerobic activity should be kept to less than 3 metabolic equivalents of task. Later, progressive aerobic exercise should be increased to 20-30 minutes, 3-5 times a week. Balance work should be incorporated. Studies on the effectiveness of exercise interventions after SARS showed benefits for endurance, maximum oxygen consumption, and strength [40].

Occupational therapy should focus on ADL and instrumental ADL guidance as well as targeted interventions to facilitate functional independence and prepare patients for discharge [41]. Speech-language pathologists should assess and treat dysphagia and voice impairments resulting from prolonged intubation and may also address respiratory strength and coordination [41]. Occupational therapists should also address cognitive changes, while speech-language pathologists should address communication issues [41]. Chinese medicine techniques such as tai chi, the Qigong 6-character mnemonic, guided breathing, and Baduanjin qigong have been suggested by the Chinese [3,38]. Education on the importance of a healthy lifestyle and participation in family and social activities should be included. Psychological interventions delivered by occupational therapists, social workers or rehabilitation psychologists may be required for patients with depression, anxiety, or PTSD [41].

## Conclusions

Rehabilitation after COVID-19 is similar to that provided for many patients in geriatric rehabilitation units who have been affected by illness or injury. Some may present with a variety of sequelae associated with the viral illness and with a prolonged stay in the ICU, possibly including mechanical ventilation. Many will have preexisting comorbidities. A thorough assessment and an individualized, progressive treatment plan which focuses on function, disability, and return to participation in society will help each patient to maximize their function and quality of life.

## Abbreviations

ADL: activity of daily living
ARDS: acute respiratory distress syndrome
CIM: critical illness myopathy
CIP: critical illness polyneuropathy
COVID-19: coronavirus disease
ICU: intensive care unit
MERS: Middle East respiratory syndrome
PPE: personal protective equipment
PTSD: posttraumatic stress disorder
SARS: severe acute respiratory syndrome
SARS-CoV-2: severe acute respiratory syndrome coronavirus 2
WHO: World Health Organization
